# Knowledge and Awareness of Dental Students and Practitioners About the Utilization of Endocrown

**DOI:** 10.3390/dj13080348

**Published:** 2025-07-29

**Authors:** Raneem Alfahad, Maryam Quritum, Lamia Hakami, Maha Aldandan, Osama Alharbi, Omar Almasoud, Abdullah Alasafirah, Passent Ellakany

**Affiliations:** 1 College of Dentistry, Imam Abdulrahman Bin Faisal University, Dammam 32210, Saudi Arabia; raneemmaher17@gmail.com (R.A.); lamiasalem11@gmail.com (L.H.); 2190003778@iau.edu.sa (M.A.); 2 Department of Pediatric Dentistry and Dental Public Health, Faculty of Dentistry, Alexandria University, Alexandria 21527, Egypt; mariam.koraytam@alexu.edu.eg; 3 College of Dentistry, Taibah University, Almadinah 42353, Saudi Arabia; osamaayidh@gmail.com; 4 College of Dentistry, King Saud University, Riyadh 4545, Saudi Arabia; on4e16@gmail.com; 5 College of Dentistry, King Faisal University, AlHoffuf 31982, Saudi Arabia; alasafirah.abdullah@gmail.com; 6 Division of Prosthodontics, School of Dentistry, University of Alabama at Birmingham, Birmingham, AL 32533, USA

**Keywords:** endocrown, student, knowledge, endodontically treated teeth, education

## Abstract

**Background/Objectives**: The aim of this study was to evaluate the level of awareness among dental students and practitioners regarding the utilization of endocrowns in clinical settings, along with any differences in knowledge based on gender, educational level, and workplace. **Methods**: A cross-sectional online survey-based study was conducted, including 1154 participants from various dental institutions across Saudi Arabia. The questionnaire included demographic data and closed-ended questions focused on knowledge and awareness of endocrowns. Data was analyzed using statistical tests, including chi-square, to see any significant differences. **Results**: Most participants (81%) had knowledge about endocrowns, mainly from their colleagues. Knowledge levels and preferences (like the use of lithium disilicate and adhesive resin cement) differed based on gender, educational level, and workplace. Male participants and undergraduates showed better awareness in some areas. Faculty members mostly depend on college training, while private practitioners obtain most of their information from workshops. **Conclusions:** Males showed significant superiority in knowledge about endocrown usage. Colleges were the most prevalent source of information regarding endocrown restorations. Significant molar tooth loss and restricted inter-arch space were the most common reasons for utilizing endocrowns. Moreover, endocrowns were considered a viable alternative to traditional post and core.

## 1. Introduction

The restoration of endodontically treated teeth is crucial. Following root canal therapy, endodontically treated teeth exhibit compromised mechanical properties, including reduced fracture resistance, flexural strength, elastic modulus, as well as moisture content, resulting in high rates of failures [1]. These teeth often suffer from significant loss of coronal structure resulting from dental caries, trauma, or previous extensive restorative procedures [2]. This makes restoring these teeth challenging and relies mainly on selecting an appropriate technique that not only ensures mechanical integrity but also maintains esthetic appearance and long-term durability [3].

Post and core restorations have long been regarded as the gold standard, especially where there has been significant coronal tooth loss, to provide prosthetic crowns with adequate retention and stability [4]. Although post and core restorations offer several advantages in terms of stability and load distribution, they have significant disadvantages as well [5]. The major disadvantages of using post and cores are the need for extensive tooth preparation and the introduction of stress concentration within the root canal system, increasing the risk of root fractures, which might result in extraction [6]. These disadvantages played a major role in searching for more conservative options to preserve the tooth structure in addition to providing acceptable function and longevity [6].

Recently, endocrowns have been considered a viable substitute, replacing traditional post and core restorations due to the elimination of the aforementioned drawbacks of posts and cores. This endocrown restoration is fabricated mainly of a monolithic ceramic restoration that combines both the core and the crown in a single prosthesis [7,8]. Additionally, endocrowns rely on adhesive bonding to the pulp chamber and the remaining coronal dentin, resulting in enhanced stability, eliminating intraradicular tooth preparation that would reduce the liability of root fracture [7,9,10].

Endocrowns are suggested for posterior teeth, particularly molars, utilizing adhesive resin cement and the pulp chamber’s interior walls to gain both micro retention and macro retention, respectively [11]. These advantages were supported by previous studies that proved that endocrowns can provide comparable mechanical performance compared to conventional restorations, with enhanced resistance to fracture and lower stress distribution within the tooth structure [6,12].

Despite these advantages, the use of endocrowns is still limited due to several postulations. First, there is a lack of standardized guidelines regarding teeth preparation and cementation, leading to variability in clinical outcomes [4]. Second, the cement and ceramic material selection is a critical factor that would affect the success and longevity of endocrown restorations; research indicates that adhesive resin cements yield the optimum bond strength and durability [4,13]. Zirconia-based endocrowns have been investigated, but because of the greater fracture resistance and the absence of specific surface treatments to improve adhesion, they are not considered the optimum choice [13]. Lithium disilicate is more favored in such cases for its strength-to-esthetic balance [12]. Moreover, there is a significant barrier to the widespread application of endocrowns among dental professionals and students [7,14]. This might be due to a gap in the curricula of dental schools with regard to covering the topic of endocrown in detail and its clinical applications [12]. That explains why the current study is being conducted—in Saudi Arabian dental institutions, research on the use and effectiveness of endocrowns remains scarce and requires more attention [1].

This study aimed to evaluate the knowledge and awareness of dental students in relation to dental practitioners regarding the use of endocrowns, identify gaps in their understanding, and provide recommendations for improving education and training in this area. By addressing these gaps, the research might enhance the clinical application of endocrowns and contribute to better patient outcomes in restorative dentistry. The first null hypothesis states that gender difference in the knowledge and awareness of utilizing endocrown restorations. The second null hypothesis states that educational level does not have a direct impact on knowledge and awareness of the utilization of endocrowns. The third null hypothesis states that the workplace does not have any influence on the knowledge and awareness of the utilization of endocrowns in the dental field.

## 2. Materials and Methods

This cross-sectional study aimed to evaluate the knowledge and awareness of the utilization of endocrowns among dental students, interns, and faculty members from Imam Abdulrahman Bin Faisal University (IAU) and various dental colleges located in Alahssa, Jeddah, Riyadh, and Almadinah Almonawarah, with a total sample size of 1154 participants. The sample size for this study was calculated using the web-based software Raosoft.Inc. Considering a dental population of more than 20,000, a margin of error of 5%, and a 95% confidence interval, the estimated sample size was 377 participants. This study employed a self-administered, online questionnaire in English, which consisted of two main sections: demographic questions and closed-ended questions focused on the participants’ knowledge of endocrowns utilization. The questionnaire platform was configured to allow only one submission per device (IP/device restriction). The online survey was designed so that all questions were mandatory, which ensured that only fully completed responses could be submitted. As a result, there was no missing data in the dataset. This study was granted ethical approval (IRB# 2024–02-616) by the Institutional Review Board of the Imam Abdulrahman Bin Faisal University, Dammam, Saudi Arabia, in 2024.

The demographic section included data on age, gender, nationality, educational institute, years of professional experience, and workplace, providing insights into the backgrounds of the respondents. Inclusion criteria specified that participants had to be at least 18 years old, either current students or graduates of a dental school, and proficient in English. Exclusion criteria eliminated students from non-dental disciplines and individuals living outside Saudi Arabia. To maximize reach, the survey was disseminated through popular platforms such as email, Facebook, Instagram, and WhatsApp, ensuring that it was accessible to a wide audience of eligible participants. An introductory statement was included to explain the purpose of the questionnaire, followed by an informed consent section to affirm that participation was voluntary, and responses would remain anonymous.

The questionnaire was validated based on a previous study from Hail University, ensuring its reliability and relevance to the research objectives [14]. Participants were questioned about their familiarity with the concepts of endocrowns, sources of information, and clinical experiences, along with specific questions regarding indications for use, advantages and disadvantages compared to conventional crowns, and various technical details associated with endocrown restorations. A pilot study was conducted to determine the time required to complete the survey and to address any challenges in comprehension of the questions. A committee of professionals reviewed the questionnaire’s English language and certified the quality and logical structure of its questions. The questionnaire was further examined by 20 dental students and dentists at various phases of their careers to examine its face validity by utilizing a 3-point Likert scale with “Yes” and “No”, and “Don’t know”, with a few clarifications made. Cohen’s Kappa test of the replies was calculated, obtaining a score of 0.87, indicating good agreement.

The closed-ended questions facilitated quantitative analysis, allowing researchers to systematically assess the knowledge levels of the participants and identify the potential gaps limiting their utilization of endocrowns.

Frequency and percentages were calculated as part of the descriptive statistics. knowledge and awareness variables were compared with the participants’ demographics. After data collection, statistical analyses were conducted to summarize the findings, exploring relationships between the demographic variables and the knowledge characteristics using a chi-square test. *p*-value less than 0.05 were considered statistically significant. Analyses were conducted using the SPSS statistical package for social sciences, Version 23.0 (SPSS Inc., Chicago, IL, USA).

## 3. Results

The questionnaire was completed by 1154 participants, with a response rate of 94%, and 60.4% of them were males. Approximately half of the participants (50.6%) were undergraduate students. The data revealed that 62.7% of the participants were employed as academics, while only 12.8% were employed in the private sector. Additionally, 75.7% of the participants had less than 5 years of professional experience, and only 11% of them had more than 10 years of professional experience (Table 1).

Table 2 shows the distribution of participants according to their knowledge and awareness of endocrowns. It was found that the majority of the participants (81%) knew about endocrowns, with the highest percentage of them (64.3%) receiving their information from college. It also revealed that 72.7% of the participants expressed that endocrown restorations are appropriate for molar teeth. Approximately half of the sample (50.9%) favored the utilization of endocrowns over the usual approach of post and core restoration, especially in cases where there is a limited amount of inter-arch space. In addition, 35.6% of the participants preferred lithium disilicate ceramic for endocrown fabrication. On the other hand, about 54.5% of them selected the adhesive resin cement as the optimal choice for the cementation of endocrown, and 48.7% admitted that the endocrown can be used as an alternative to conventional post and core.

Table 3 shows that there were significant associations between gender and different variables of knowledge and awareness about endocrown (source of information they obtain their knowledge from, usage of endocrown, the preferred material of fabrication of endocrown, and the type of cement used for endocrown).

Regarding the source of information, the college was selected as the source of knowledge among male participants with a significant difference compared to females (65.3%, 62.7%, *p* = 0.04). Also, a significantly higher percentage of males reported that endocrowns are used for molars than females (77.9%, 64.8%, *p* < 0.001). In addition, a significantly higher percentage of males preferred lithium disilicate for the fabrication of endocrowns than females (38.6%, 31,1%, *p* = 0.002), as well as the adhesive resin cement for cementation of endocrowns (59.5%, 46.8%, *p* < 0.001).

Table 4 reveals that there were significant associations between all variables of knowledge and awareness about endocrown utilization among participants of different educational levels. College was the most common source of knowledge about endocrown restorations among the different groups of participants. Molars, extensive loss of tooth structure, and limited inter-arch space were the most favorable situations in using endocrown restorations among all groups of participants. Lithium disilicate and adhesive resin cements were selected by most participants in different studied groups to fabricate and bond endocrown restorations to tooth structure. Most of the participants from the tested groups chose endocrown as a viable substitute for conventional post and core, except for the group of specialized participants.

Data in Table 5 shows that there were significant associations between workplace of participants with different variables of knowledge and awareness about endocrown utilization which included where participants obtained knowledge about endocrowns (*p* < 0.001), usage of endocrown restorations (*p* = 0.004), the condition that favored the utilization of endocrowns over the standard approach of post and core restoration (*p* = 0.02), the preferred material used in the fabrication of endocrowns (*p* < 0.001), and finally considering the usage of endocrown as an acceptable alternative to conventional post and core (*p* = 0.004).

## 4. Discussion

Endocrowns could be considered as a viable substitute for restoring endodontically treated molars that underwent significant structural tooth loss [6,14]. The results showed that male participants relied mainly on their college education as the main source of information about endocrown restorations more than females. Moreover, a far greater proportion of males than females stated that endocrown is used mainly for restoring endodontically treated molars with extensive tooth loss and in cases of limited inter-arch space. There were significant associations between all variables of knowledge and awareness about endocrown utilization among participants of different educational levels. However, college was cited by the different participant groups as the most common source of information regarding endocrown restorations. Significant relationships were found between the participants’ workplace and various endocrown knowledge and awareness variables, such as the participants’ source of information, their use of endocrown restoration, the conditions that encouraged the use of endocrown over the conventional post-and-core restoration method, the material of choice for endocrown fabrication, and the possibility that endocrown could replace traditional post-and-core restoration. Thus, the three hypotheses were rejected.

Material preferences also varied by gender: males preferred lithium disilicate in the fabrication of endocrown restorations nearly 40% of the time, compared to 31% among females. Additionally, 60% of male participants favored adhesive resin cement for cementation, compared to 47% of females. These results align with Madfa et al. [15], who agreed on using lithium disilicate ceramics (55.1%) and resin cement (85.3%) in endocrown restorations irrespective of contextual factors. Additionally, the use of lithium disilicate ceramics in the fabrication of endocrowns was supported by multiple studies because of its advantageous mechanical qualities, esthetically pleasing results, and the capacity to adhere to the tooth structure [16,17,18,19]. When compared to other materials, it has the highest fracture resistance, particularly when subjected to lateral force. However, other research approved the utilization of resin composite endocrown due to the low elastic modulus of resin composite materials, which is comparable to that of dentin, leading to the introduction of composite as an alternative to ceramic materials in endocrown fabrication [6,16,17,18]. More advantageous modes of failure are the consequence of the similarity in properties between resin composite and natural dentin, resulting in a proper stress distribution [18,19,20].

Resin composites can also be shaped and repaired intraorally, unlike ceramics [10]. Additionally, resin composites offer higher fracture resistance, compared to other ceramic materials, according to several studies [1,6,18]. Because of their superior bonding strength, good mechanical qualities, low solubility, and esthetic appeal, resin cement is frequently employed for endocrown cementation [12,18,21]. On the other hand, resin composite has some drawbacks, for example, they typically exhibit a greater degree of marginal leakage [1,6,18]. The clinical success of the endodontic treatment depends mainly on the adhesive approach, which stops marginal leakage and lessens the invasion of bacteria from the crown toward the apex [6].

Workplace setting significantly influenced participants’ knowledge and attitudes toward endocrowns. Academic dentists were more likely to obtain the information from the college (65.6%), while private practitioners favored conferences and workshops (14.4%). Most of the participants used endocrowns frequently in restoring molar teeth and limited inter-arch space conditions. Limited inter-arch space was reported in the literature as one of the indications of endocrown usage [22]. Moreover, lithium disilicate ceramics are preferred in the fabrication of endocrowns mainly by academics. This agrees with the findings of a published systematic review [6]. Notably, almost half of the participants believed endocrowns could replace conventional post and core restorations [10]. Supporting studies similarly found consensus on material and cement preferences, though adoption rates varied due to differences in sample size, culture, and educational exposure [15,21].

While Madfa et al. [15] reported a high awareness rate of endocrowns (93.1%), our study revealed a slightly lower level (81%), which may be attributed to the broader demographic variability or regional differences in training quality [18,19,20]. Moreover, Madfa et al. [15] identified workplace environment as the primary factor influencing awareness (*p* < 0.05). This comes in contrast with our findings, which suggested that educational background might have a more substantial and lasting impact on knowledge retention.

The present study’s strengths included the utilization of a large and diverse sample enabled more nuanced analysis through stratification by educational level and workplace setting, and underscored the pivotal role of educational institutions in shaping foundational knowledge about endocrowns. Additionally, it might provide a new layer of understanding by highlighting these gender differences in endocrown restoration usage and knowledge. Discrepancies among the current findings highlighted the need for specific educational strategies and reinforced the importance of enhancing postgraduate training programs. As emphasized in the systematic review, standardized and comprehensive education is essential to ensure consistent, evidence-based clinical practice in restorative dentistry [6].

However, the limitations were having a cross-sectional and self-reported nature, as well as lacking clinical skill assessments [4,9,15,23]. Cross-sectional studies can provide information about the prevalence of knowledge among participants without providing a causal relationship between tested variables, in addition to the susceptibility to bias that might be encountered in the findings. Furthermore, the questionnaire was limited to participants living in Saudi Arabia, so the findings cannot be generalized with a potential selection bias and the use of a non-randomized convenience sample. Workplace environment and educational exposure both play critical roles in shaping endocrown awareness and clinical decision-making, underscoring the need for standardized, evidence-based training across practice settings. Further studies are required to assess the knowledge and usage of endocrown restorations in different countries compared to Saudi Arabia. Moreover, evaluating the students’ and practitioners’ knowledge and usage of CAD/CAM endocrown restorations compared to conventional endocrowns is required. Future research should focus on evaluating clinical competency and long-term outcomes associated with endocrown usage to support its integration into routine dental practice.

## 5. Conclusions

This study highlights notable differences in knowledge and awareness regarding endocrowns among dental students and practitioners in Saudi Arabia. Males showed significant superiority in knowledge and usage of endocrowns over females. Among the various participant groups, college was the most prevalent source of information regarding endocrown repairs. Significant molar tooth loss and restricted inter-arch space were the most common reasons for utilizing endocrown restorations. The majority of participants chose to construct and bond endocrown restorations to the tooth structure using lithium disilicate and adhesive resin cements. Moreover, endocrown was considered a viable alternative to traditional post and core. These findings underscore the importance of having well-structured curricula in dental schools, superimposed with evidence-based training on endocrowns in both undergraduate and postgraduate dental programs. Enhancing the curriculum and offering targeted workshops can help bridge existing knowledge gaps and encourage broader clinical adoption of endocrowns, ultimately improving restorative outcomes for patients.

## Figures and Tables

**Table 1:** Distribution of subjects based on socio-demographic characteristics (Total 1154).

Variables		Frequency	Percentage
Gender	Male	697	60.4%
	Female	457	39.6%
Education	Undergraduate	584	50.6%
	Intern	249	21.6%
	General practitioner	163	14.1%
	Resident	68	5.9%
	Specialties *	90	7.8%
Workplace/affiliation	Academic	723	62.7%
	Governmental	283	24.5%
	Private	148	12.8%
Years of experience	<5 years	874	75.7%
	5–10 years	153	13.3%
	>10 years	127	11%

* Specialties: prosthodontist/restorative dentist/faculty and other specialties.

**Table 2:** Distribution of subjects based on knowledge and awareness about endocrown, created by the author.

Variables		Frequency	Percentage
Are you knowledgeable about the concept of endocrown?	Yes	935	81%
	No	219	19%
If yes, where did you obtain the information?	College	601	64.3%
	Friends	97	10.4%
	Textbook	99	10.6%
	Internet	94	10.1%
	Conference and workshop	44	4.7%
The endocrown restoration is used for	Anterior teeth	102	8.8%
	Molars	839	72.7%
	Premolars	62	5.4 %
	Don’t know	151	13.1%
What is the indication of using endocrowns?	Extensive loss of tooth structure	505	43.8%
	Moderate loss of tooth structure	214	18.5%
	Minimum loss of tooth structure	250	21.7%
	Don’t know	185	16%
In which clinical scenario is the utilization of endocrown favored above the standard approach of post and core restoration?	Limited inter-arch space	587	50.9%
	Enough inter-arch space	328	28.4%
	Don’t know	239	20.7%
Do you need a ferrule effect for endocrowns?	Yes	475	41.2%
	No	325	28.2%
	Don’t know	354	30.7%
The preferred material for the fabrication of endocrowns	Zirconia	279	24.2%
	Lithium disilicate	411	35.6%
	Nanocomposite resin	110	9.5%
	Fieldspathic porcelain	63	5.5%
	Don’t know	291	25.2%
What type of cement is used for endocrowns?	Adhesive resin cement	629	54.5%
	Glass ionomer	119	10.3%
	Zinc phosphate	114	9.9%
	Don’t know	292	25.3%
Can endocrowns be an alternative to conventional posts and cores?	Yes	562	48.7%
	No	227	19.7%
	Don’t know	365	31.6%

**Table 3:** Association of knowledge and awareness about endocrown with the gender of the participants.

Variables		Gender N (%)		
		Male	Female	*p* Value
Are you knowledgeable about the concept of endocrown?	Yes	568 (81.5)	367 (80.3)	0.62
	No	129 (18.5)	90 (19.7)	
If yes, where did you obtain the information?	College	371 (65.3)	230 (62.7)	0.04
	Friends	47 (8.3)	50 (13.6)	
	Textbook	68 (12)	31 (8.4)	
	Internet	58 (10.2)	36 (9.8)	
	Conference and workshop	24 (4.2)	20 (5.4)	
The endocrown restoration is used for:	Anterior teeth	47 (6.7)	55 (12)	<0.001
	Molars	543 (77.9)	296 (64.8)	
	Premolars	28 (4)	34 (7.4)	
	Don’t know	79 (11.3)	72 (15.8)	
What is the indication of using endocrowns?	Extensive loss of tooth structure	298 (42.8)	207 (45.3)	0.14
	Moderate loss of tooth structure	124 (17.8)	90 (19.7)	
	Minimum loss of tooth structure	167 (24)	83 (18.2)	
	Don’t know	108 (15.5)	77 (16.8)	
In which clinical scenario is the utilization of endocrown favored above the standard approach of post and core restoration?	Limited inter-arch space	361 (51.8)	226 (49.5)	0.61
	Enough inter-arch space	198 (28.4)	130 (28.4)	
	Don’t know	138 (19.8)	101 (22.1)	
Do you need a ferrule effect for endocrowns?	Yes	292 (41.9)	183 (40)	0.79
	No	192 (27.5)	133 (29.1)	
	Don’t know	213 (30.6)	141 (30.9)	
The preferred material for the fabrication of endocrowns	Zirconia	173 (24.8)	106 (23.2)	0.002
	Lithium disilicate	269 (38.6)	142 (31.1)	
	Nanocomposite resin	49 (7)	61 (13.3)	
	Fieldspathic porcelain	34 (4.9)	29 (6.3)	
	Don’t know	172 (24.7)	119 (26)	
What type of cement is used for endocrowns?	Adhesive resin cement	415 (59.5)	214 (46.8)	<0.001
	Glass ionomer	55 (7.9)	64 (14)	
	Zinc phosphate	55 (7.9)	59 (12.9)	
	Don’t know	172 (24.7)	120 (26.3)	
Can endocrowns be an alternative to conventional posts and cores?	Yes	335 (48.1)	227 (49.7)	0.12
	No	127 (18.2)	100 (21.9)	
	Don’t know	235 (33.7)	130 (28.4)	

**Table 4:** Association of knowledge and awareness about endocrown with the education of the participants.

Variables		Education N (%)					
		Undergraduate	Intern	General Practitioner	Resident	Specialties	*p* Value
Are you knowledgeable about the concept of endocrown?	Yes	481 (82.4)	199 (79.9)	141 (86.5)	55 (80.9)	59 (65.6)	0.001
	No	103 (17.6)	50 (20.1)	22 (13.5)	13 (19.1)	31 (34.4)	
If yes, where did you obtain the information?	College	348 (72.3)	124 (62.3)	73 (51.8)	32 (58.2)	24 (40.7)	<0.001
	Friends	47 (9.8)	24 (12.1)	12 (8.5)	7 (12.7)	7 (11.9)	
	Textbook	34 (7.1)	21 (10.6)	22 (15.6)	9 (16.4)	13 (22)	
	Internet	50 (10.4)	19 (9.5)	15 (10.6)	4 (7.3)	6 (10.2)	
	Conference and workshop	2 (0.4)	11 (5.5)	19 (13.5)	3 (5.5)	9 (15.3)	
The endocrown restoration is used for	Anterior teeth	54 (9.2)	20 (8)	10 (6.1)	12 (17.6)	6 (6.7)	<0.001
	Molars	395 (67.6)	213 (85.5)	132 (81)	43 (63.2)	56 (62.2)	
	Premolars	29 (5)	13 (5.2)	11 (6.7)	6 (8.8)	3 (3.3)	
	Don’t know	106 (18.2)	3 (1.2)	10 (6.1)	7 (10.3)	25 (27.8)	
What is the indication of using endocrowns?	Extensive loss of tooth structure	247 (42.3)	129 (51.8)	67 (41.1)	28 (41.2)	34 (37.8)	<0.001
	Moderate loss of tooth structure	103 (17.6)	41 (16.5)	38 (23.3)	17 (25)	15 (16.7)	
	Minimum loss of tooth structure	115 (19.7)	63 (25.3)	41 (25.2)	16 (23.5)	15 (16.7)	
	Don’t know	119 (20.4)	16 (6.4)	17 (10.4)	7 (10.3)	26 (28.9)	
In which clinical scenario is the utilization of endocrown favored above the standard approach of post and core restoration?	Limited inter-arch space	270 (46.2)	161 (64.7)	81 (49.7)	37 (54.4)	38 (42.2)	<0.001
	Enough inter-arch space	167 (28.6)	68 (27.3)	54 (33.1)	20 (29.4)	19 (21.1)	
	Don’t know	147 (25.2)	20 (8)	28 (17.2)	11 (16.2)	33 (36.7)	
Do you need a ferrule effect for endocrowns?	Yes	234 (40.1)	107 (43)	75 (46)	29 (42.6)	30 (33.3)	<0.001
	No	125 (21.4)	108 (43.4)	49 (30.1)	21 (30.9)	22 (24.4)	
	Don’t know	225 (38.5)	34 (13.7)	39 (23.9)	18 (26.5)	38 (42.2)	
The preferred material for the fabrication of endocrowns	Zirconia	141 (24.1)	57 (22.9)	45 (27.6)	15 (22.1)	21 (23.3)	<0.001
	Lithium disilicate	166 (28.4)	129 (51.8)	67 (41.1)	24 (35.3)	25 (27.8)	
	Nanocomposite resin	57 (9.8)	22 (8.8)	11 (6.7)	12 (17.6)	8 (8.9)	
	Fieldspathic porcelain	28 (4.8)	9 (3.6)	14 (8.6)	6 (8.8)	6 (6.7)	
	Don’t know	192 (32.9)	32 (12.9)	26 (16)	11 (16.2)	30 (33.3)	
What type of cement is used for endocrowns?	Adhesive resin cement	265 (45.4)	179 (71.9)	103 (63.2)	36 (52.9)	46 (51.1)	<0.001
	Glass ionomer	57 (9.8)	30 (12)	17 (10.4)	8 (11.8)	7 (7.8)	
	Zinc phosphate	63 (10.8)	18 (7.2)	18 (11)	9 (13.2)	6 (6.7)	
	Don’t know	199 (34.1)	22 (8.8)	25 (15.3)	15 (22.1)	31 (34.4)	
Can endocrowns be an alternative to conventional posts and cores?	Yes	248 (42.5)	167 (67.1)	79 (48.5)	36 (52.9)	32 (35.6)	<0.001
	No	113 (19.3)	41 (16.5)	36 (22.1)	17 (25)	20 (22.2)	
	Don’t know	223 (38.2)	41 (16.5)	48 (29.4)	15 (22.1)	38 (42.2)	

**Table 5:** Association of knowledge and awareness about endocrown with the workplace of the participants.

Variables		Workplace			
		Academic	Governmental	Private	*p* Value
Are you knowledgeable about the concept of endocrown?	Yes	585 (80.9)	239 (84.5)	111 (75)	0.06
	No	138 (19.1)	44 (15.5)	37 (25)	
If yes, where did you obtain the information?	College	384 (65.6)	163 (68.2)	54 (48.6)	<0.001
	Friends	68 (11.6)	15 (6.3)	14 (12.6)	
	Textbook	60 (10.3)	24 (10)	15 (13.5)	
	Internet	60 (10.3)	22 (9.2)	12 (10.8)	
	Conference and workshop	13 (2.2)	15 (6.3)	16 (14.4)	
The endocrown restoration is used for	Anterior teeth	58 (8)	19 (6.7)	25 (16.9)	0.004
	Molars	536 (74.1)	211 (74.6)	92 (62.2)	
	Premolars	32 (4.4)	19 (6.7)	11 (7.4)	
	Don’t know	97 (13.4)	34 (12)	20 (13.5)	
What is the indication of using endocrowns?	Extensive loss of tooth structure	313 (43.3)	128 (45.2)	64 (43.2)	0.82
	Moderate loss of tooth structure	131 (18.1)	55 (19.4)	28 (18.9)	
	Minimum loss of tooth structure	156 (21.6)	57 (20.1)	37 (25)	
	Don’t know	123 (17)	43 (15.2)	19 (12.8)	
In which clinical scenario is the utilization of endocrowns favored above the standard approach of post and core restoration?	Limited inter-arch space	389 (53.8)	138 (48.8)	60 (40.5)	0.02
	Enough inter-arch space	187 (25.9)	85 (30)	56 (37.8)	
	Don’t know	147 (20.3)	60 (21.2)	32 (21.6)	
Do you need a ferrule effect for endocrowns?	Yes	297 (41.1)	119 (42)	59 (39.9)	0.51
	No	193 (26.7)	87 (30.7)	45 (30.4)	
	Don’t know	233 (32.2)	77 (27.2)	44 (29.7)	
The preferred material for the fabrication of endocrowns	Zirconia	164 (22.7)	78 (27.6)	37(25)	<0.001
	Lithium disilicate	275 (38)	89 (31.4)	47 (31.8)	
	Nanocomposite resin	64 (8.9)	20 (7.1)	26 (17.6)	
	Fieldspathic porcelain	36 (5)	13 (4.6)	14 (9.5)	
	Don’t know	184 (25.4)	83 (29.3)	24 (16.2)	
What type of cement is used for endocrowns?	Adhesive resin cement	408 (56.4)	145 (51.2)	76 (51.4)	0.06

## Data Availability

Data supporting the reported results is available upon request from the principal due to privacy or ethical restrictions.
